# Supplementary material for: Mitochondrial H2O2 release does not directly cause damage to chromosomal DNA
Source: Nat Commun. 2024 Mar 28;15:2725. doi: 10.1038/s41467-024-47008-x (PMC10978998; doi:10.1038/s41467-024-47008-x)
Supplement: Supplementary file 3 — Description of Additional Supplementary Files [file 41467_2024_47008_MOESM3_ESM.pdf]

Description of Additional Supplementary Files

File Name: Supplementary Movie 1
Description: (cropped stills can be found in Supplementary Fig. 13, upper panel). RPE-hTERT-DAAOH2B cells expressing the FUCCI cell cycle marker were imaged for 25.5 h in the presence of 10 mM L-Ala Cells in G0/1 phase are indicated in blue (mKO2-CDT1) and cells in in S/G2 phase in green (mAG1-Gemenin). In the presence of L-Ala, RPE1-hTERT DAAOH2B cells transition normally from G2 (green) to mitosis (colorless), resulting in the appearance of 2 blue daughter cells. Scale bar = 60 µm.

File Name: Supplementary Movie 2
Description: (cropped stills can be found in Supplementary Fig. 13, lower panel). RPE-hTERT-DAAOH2B cells expressing the FUCCI cell cycle marker were imaged for 25.5 h in the presence of 10 mM D-Ala. Cells in G0/1 phase are indicated in blue (mKO2- CDT1) and cells in in S/G2 phase in green (mAG1-Gemenin). In the presence of D-Ala, green RPE1-hTERT-DAAOH2B (G2) transition directly to blue (G0/1) without dividing, indicative of a mitotic bypass. Scale bar = 60 µm.
