# Supplementary material for: Mitochondrial H2O2 release does not directly cause damage to chromosomal DNA
Source: Nat Commun. 2024 Mar 28;15:2725. doi: 10.1038/s41467-024-47008-x (PMC10978998; doi:10.1038/s41467-024-47008-x)
Supplement: Supplementary file 1 — Supplementary Information [file 41467_2024_47008_MOESM1_ESM.pdf]

Supplementary information related to:

Mitochondrial H_2O_2 release does not directly cause damage to chromosomal DNA.

Daan M.K. van Soest[1], Paulien E. Polderman[1], Wytze T. F. den Toom[1], Janneke P. Keijer[1], Markus J. van Roosmalen[2], Tim M. F. Leyten[1], Johannes Lehmann[1], Susan Zwakenberg[1], Sasha de Henau[1], Ruben van Boxtel[2,3], Boudewijn M. T. Burgering[1,3], Tobias B. Dansen[1@]

Affiliations
1 Center for Molecular Medicine, University Medical Center Utrecht, Universiteitsweg 100, 3584 CG Utrecht, The Netherlands
2 Princess Máxima Center for Pediatric Oncology, Heidelberglaan 25, 3584 CS, Utrecht, the Netherlands.
3 Oncode Institute, Jaarbeursplein 6, 3521 AL Utrecht, The Netherlands.@corresponding author:
@ t.b.dansen@umcutrecht.nl

Contents:
Sixteen Supplemental Figures including legends.

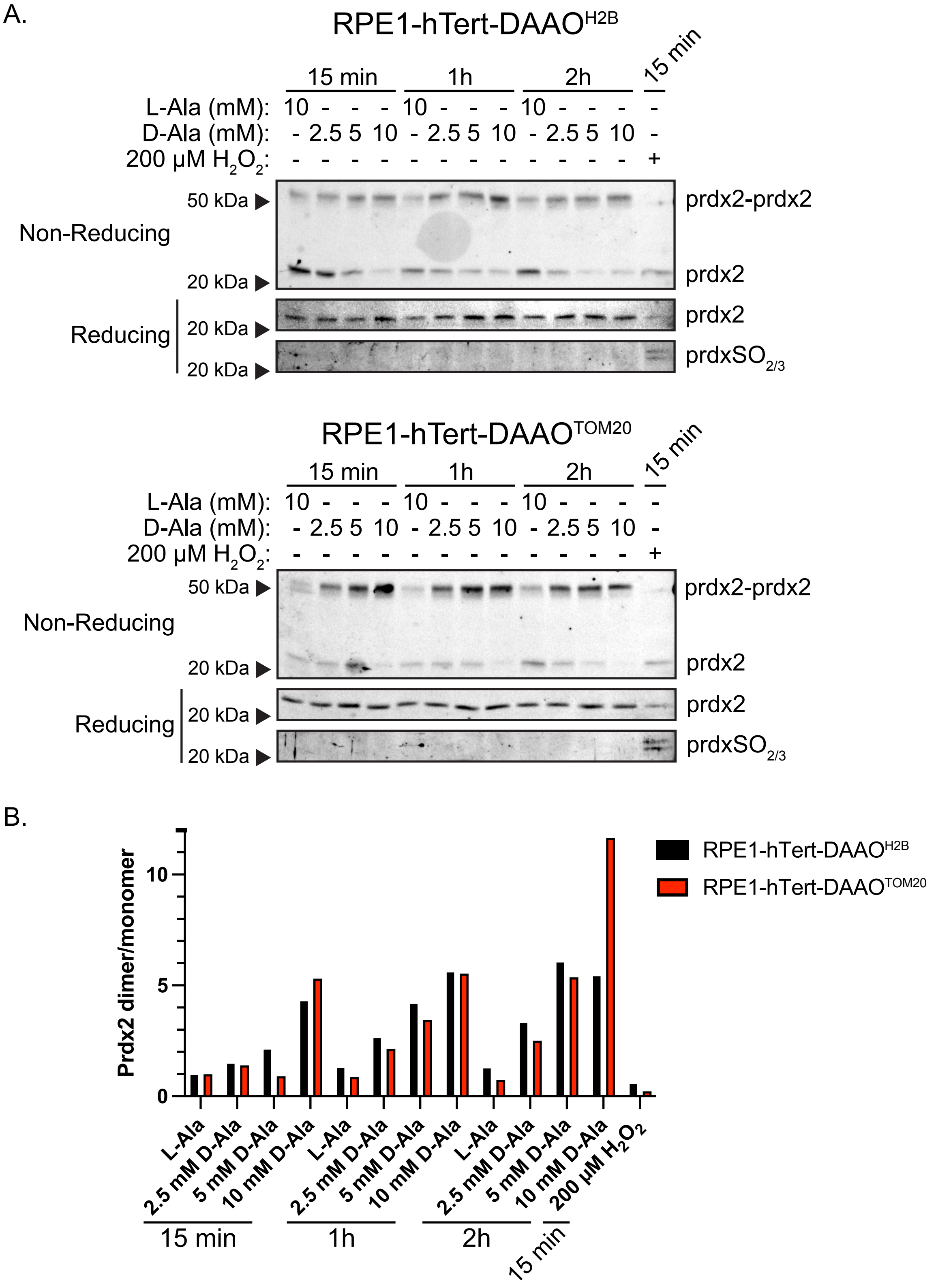

Supplementary figure 1. DAAO-produced H_2O_2 induces PRDX2 dimerization but not overoxidation. (a) Non-reducing and reducing Western blots for PRDX2 and PRDX-$SO_{2/3}$ in RPE1-hTERT-DAAO^{H2B} and RPE1-hTERT-DAAO^{TOM20} cells. DAAO activation results in PRDX2 oxidation and subsequent dimerization in a concentration dependent manner, but does not result in PRDX overoxidation (PRDX-$SO_{2/3}$), in contrast to exogenous H_2O_2 treatment. The increase in PRDX2 dimerization upon DAAO activation has been observed several times, although dimerization at basal levels varies considerably between experiments. **(b)** Quantification of non-reducing blot shown in **(a)** Values are normalized to 15 min L-Ala condition. Source data are provided in the Associated Source Data file.

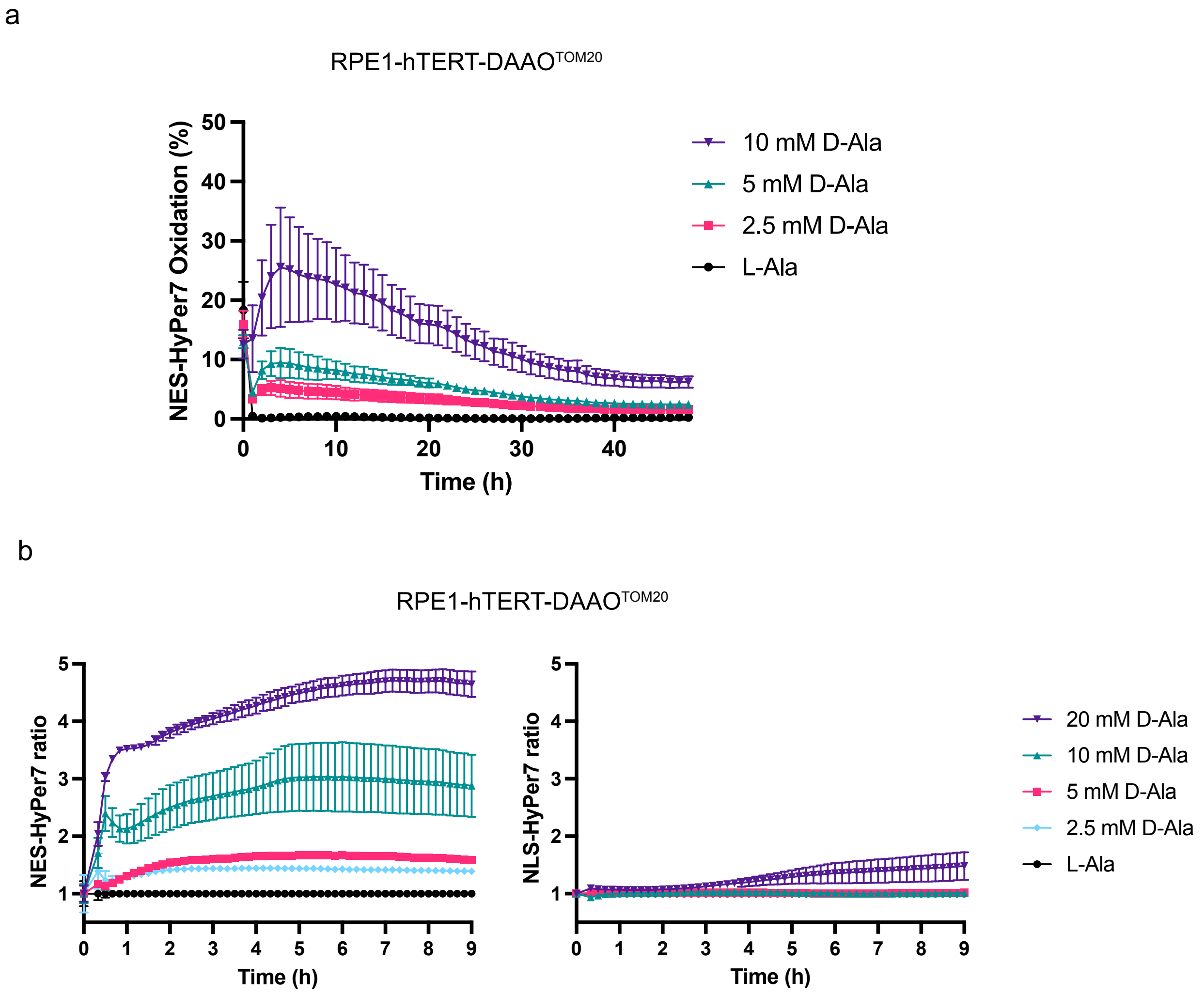

Supplementary figure 2. HyPer7 oxidation in response to DAAO activation peaks at ~ 5 hours and slowly decreases over time. (a) NES-HyPer7 measurements in RPE-hTERT-DAAOTOM20 cells upon treatment with L-Ala or D-Ala over 48h. HyPer7 oxidation increases up until 4-5 h after D-Ala injection, after which it slowly decreases over time. HyPer7 oxidation in response to 200 μM H_2O_2 exogenous was set to 100%, baseline oxidation was considered 0%. Date are presented as mean values of 4 biological repeats -/+ SD. Source data are provided in the Associated Source Data file. **(b)** NES-HyPer7 and NLS-HyPer7 measurements in RPE-hTERT-DAAO^{TOM20} cells, demonstrating that 20 mM D-Ala treatment can result in a modest increase nuclear HyPer7 oxidation after several hours. HyPer7 ratio was first normalized to the L-Ala condition, and subsequently to timepoint 0. Data are presented as mean values of 3 biological repeats -/+ SD. Source data are provided in the Source Data file.

Supplementary figure 3 (*on next page*). Absence of nuclear HyPer7 oxidation in response to H_2O_2 released from mitochondria is homogeneous over all cells. Representative images of the HyPer7 measurements quantified in main figure 2 **(a-d)**. Images display HyPer7 oxidation upon treatment with 10 mM L-Ala or D-Ala. While NES-HyPer7 oxidation is increased in nearly al RPE-hTERT-DAAO^{TOM20} cells upon 10 mM D-Ala treatment, no increase of NLS-HyPer7 is observed in any cell. In contrast, NLS-HyPer7 oxidation is clearly visible in RPE-hTERT-DAAO^{H2B} cells. Scale bar: 400 μm.

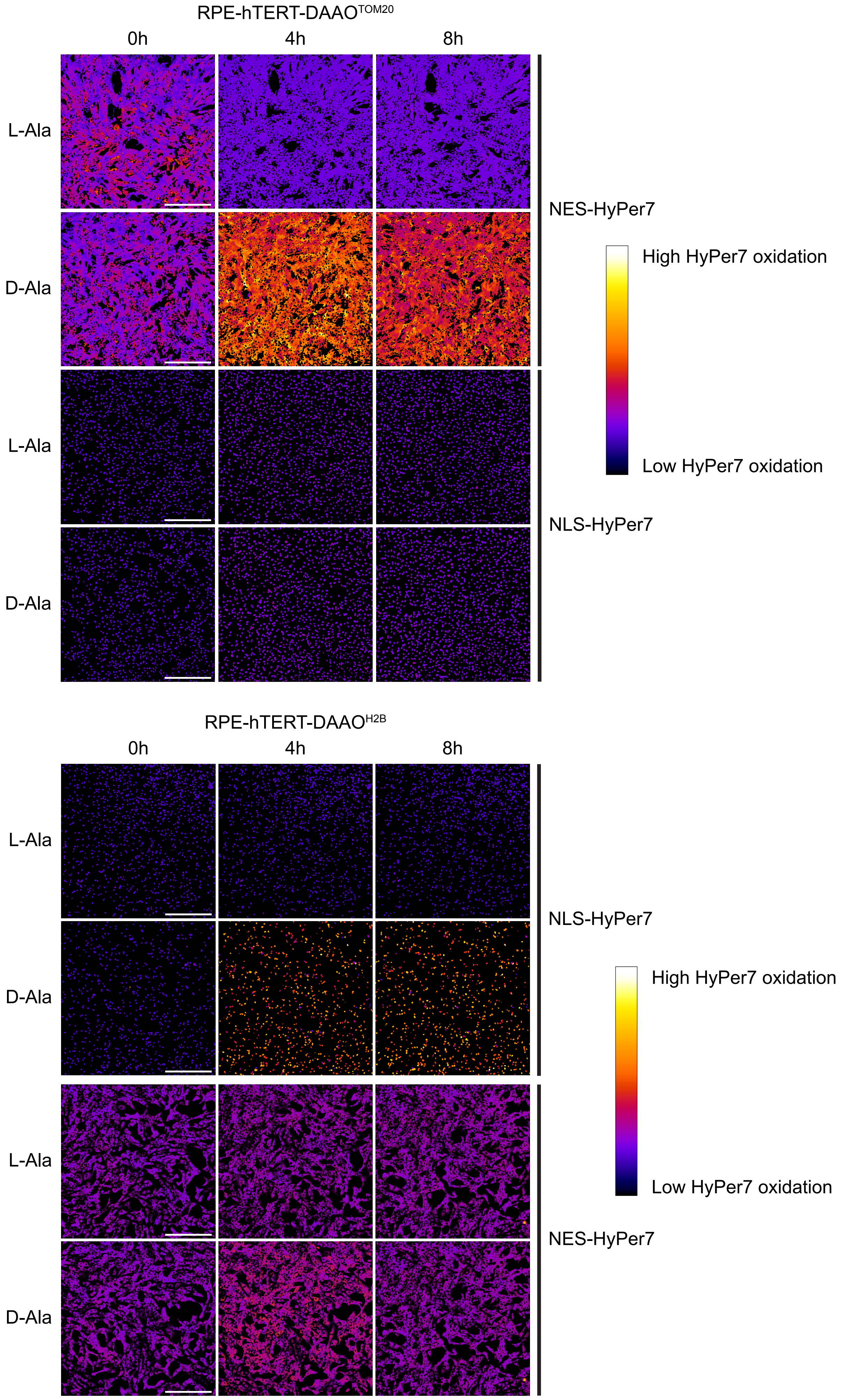

Supplementary Fig 3, *legends on previous page*

a

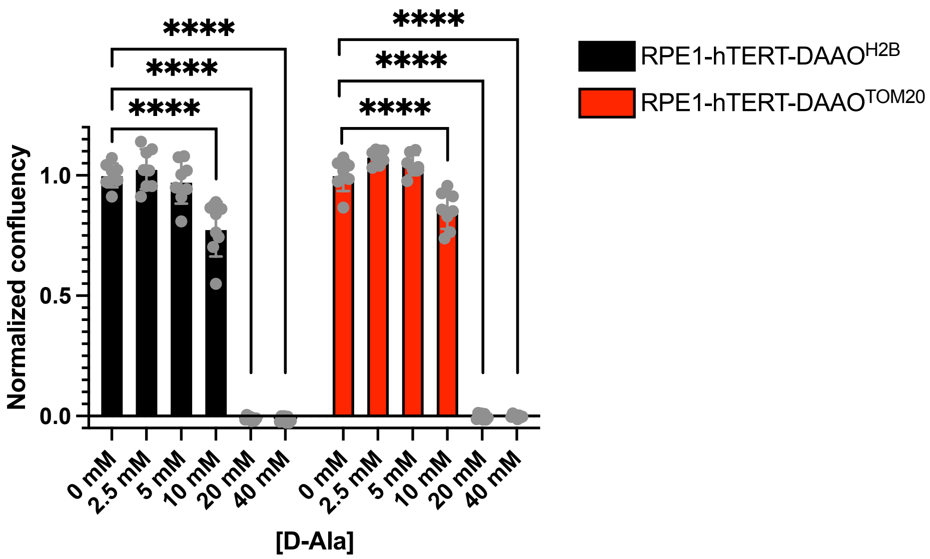

b

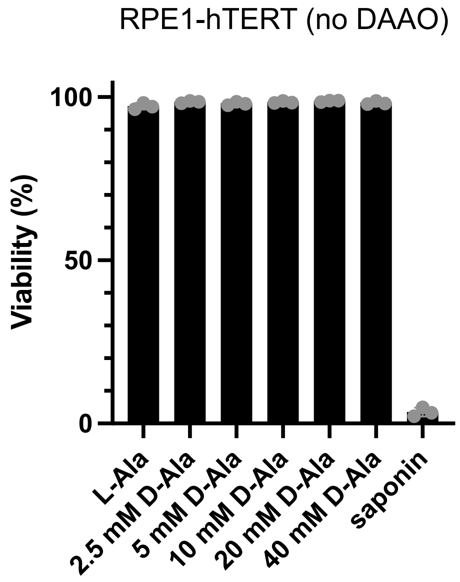

Supplementary figure 4. Viability upon DAAOH2B and DAAOTOM20 activation. (a) RPE1-hTERT-DAAO^{H2B} and RPE1-hTERT-DAAO^{TOM20} cells were treated for 48h with several concentrations of D-Ala, fixed and stained with crystal violet, followed by dissolution in 10% acetic acid and quantification by spectrophotometry. Massive cell death occurs from 20 mM D-Ala treatment in both lines. Data represent mean values of 8 individual biological repeats -/+ SD. Statistical significance was determined by ANOVA + Dunnett's multiple comparison test (**** = p≤0.0001). Source data are provided in the Source Data file. **(b)** D-Ala does not induce toxicity in parental RPE1-hTERT cells not expressing DAAO. Cell viability was measured by PI-exclusion in the parental RPE1-hTERT cells upon treatment with L-Ala or D-Ala for 48 h. No decrease in cell viability was detected up until 40 mM D-Ala treatment. Saponin treatment served as positive control for loss of membrane integrity. Data represent mean values of 3 biological repeats -/+ SD. Source data are provided in the Source Data file.

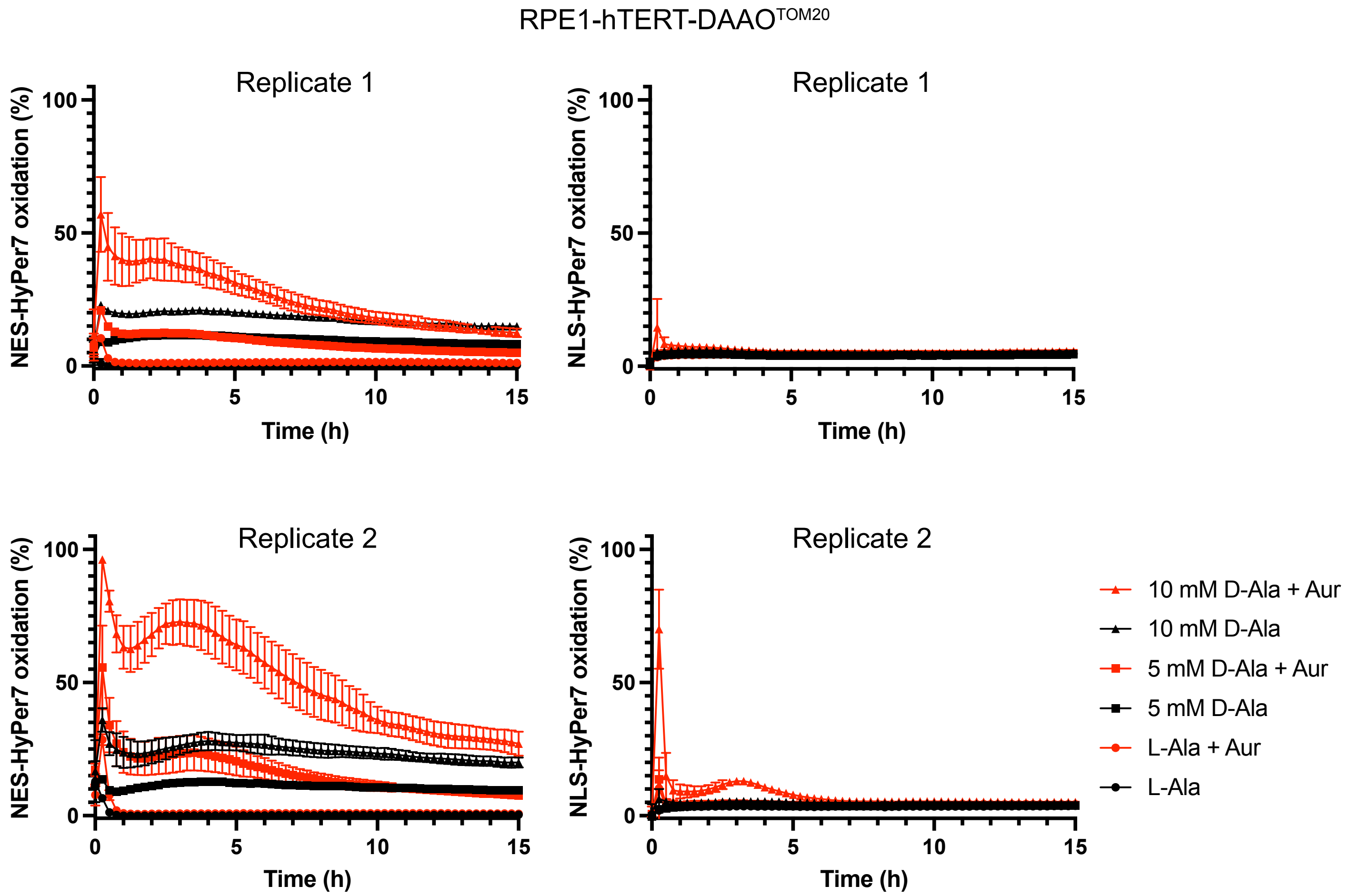

Supplementary figure 5. Supraphysiological H_2O_2 production at mitochondria does not reach the nucleus in the presence of Auranofin. NES-HyPer7 and NLS-HyPer7 measurements in RPE-hTERT-DAAO^{TOM20} cells upon treatment with L-Ala or D-Ala in the absence or presence of the Thioredoxin reductase inhibitor of Auranofin (Aur, 1 µM). Two independent experiments are shown (**upper** vs. **lower** graphs). Auranofin increases NES-HyPer7 oxidation in response to D-Ala treatment. While there is some variability in the extent of HyPer7 oxidation between experiments, in both experiments supraphysiological H_2O_2 production upon administration of 5 mM D-ala does not result in increased NLS-HyPer7 oxidation compared to L-Ala treatment in the presence of Auranofin. Higher amounts of H_2O_2 production by DAAO^{TOM20} by administration of 10 mM D-Ala is temporally detected in the nucleus in the presence of Auranofin. Data represent mean values of 2 separate wells -/+ SD. Source data are provided in the Source Data file.

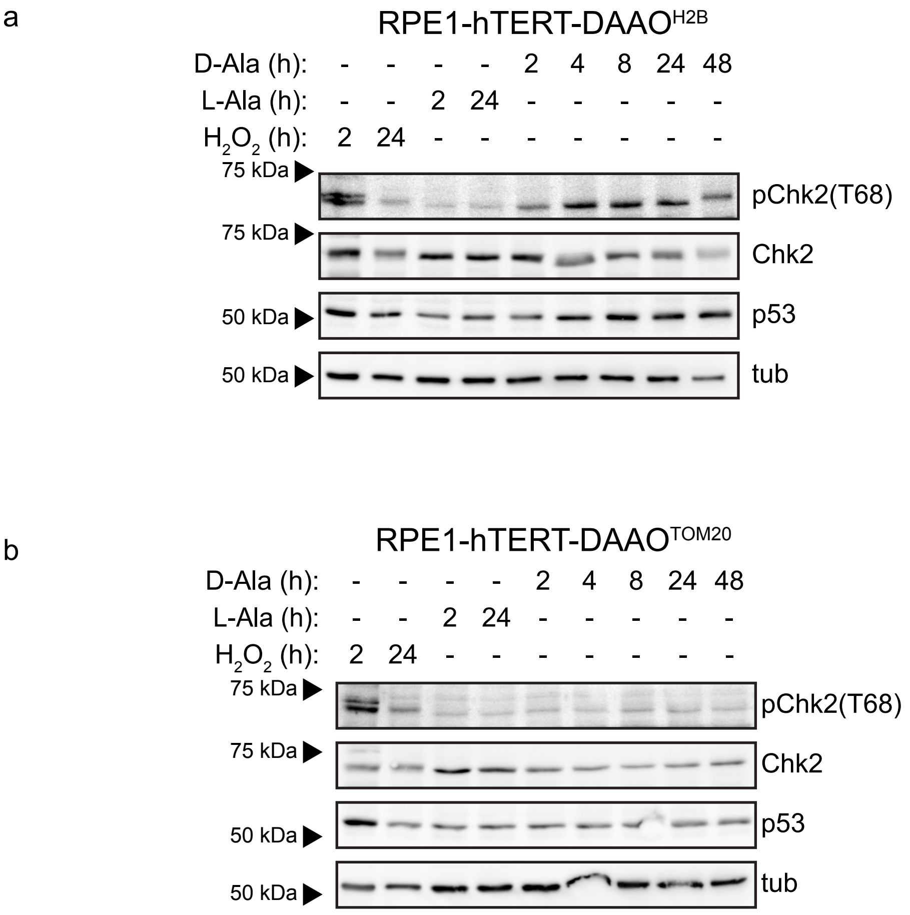

Supplementary figure 6. DDR signaling is rapidly induced after DAAO^{H2B} activation. Western blots of **(a)** RPE1-hTERT-DAAO^{H2B} and **(b)** RPE1-hTERT-DAAO^{TOM20} cells treated with L-Ala or D-Ala (15 mM) for several time points. Exogenous treatment with 200 μM H_2O_2 serves as positive control for DDR activation as measured by CHK2 phosphorylation. Already after 2h of D-Ala addition pCHK2(T68) is elevated in RPE1-hTERT-DAAO^{H2B} cells, which increases further and is sustained over the course of 48h. p53 stabilization is observed after 4h. No activation of the DNA damage response is observed in RPE1-hTERT-DAAO^{TOM20} cells upon D-Ala addition. Experiment is repeated at least three times, a representative experiment is shown. Source data are provided in the Source Data file.

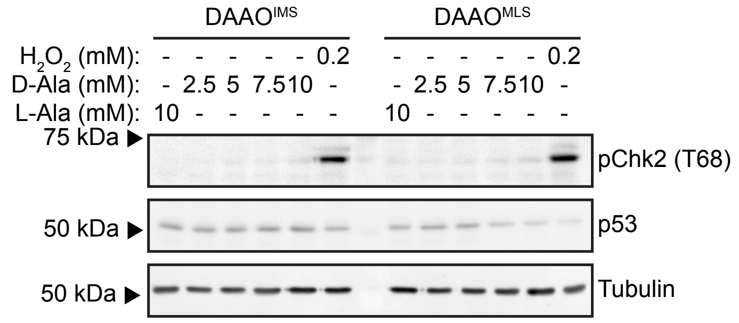

Supplementary figure 7. Intramitochondrial H_2O_2 production does not activate the DNA damage response. WB of RPE1-hTERT-DAAO^{IMS} and RPE1-hTERT-DAAO^{MLS} cells treated for 24h with L- or D-Ala. Activation of DAAO^{IMS} or DAAO^{MLS} does not result in activation of the DNA damage response. A 15 min exogenous H_2O_2 treatment was used as positive control for CHK2 phosphorylation. Source data are provided in the Source Data file.

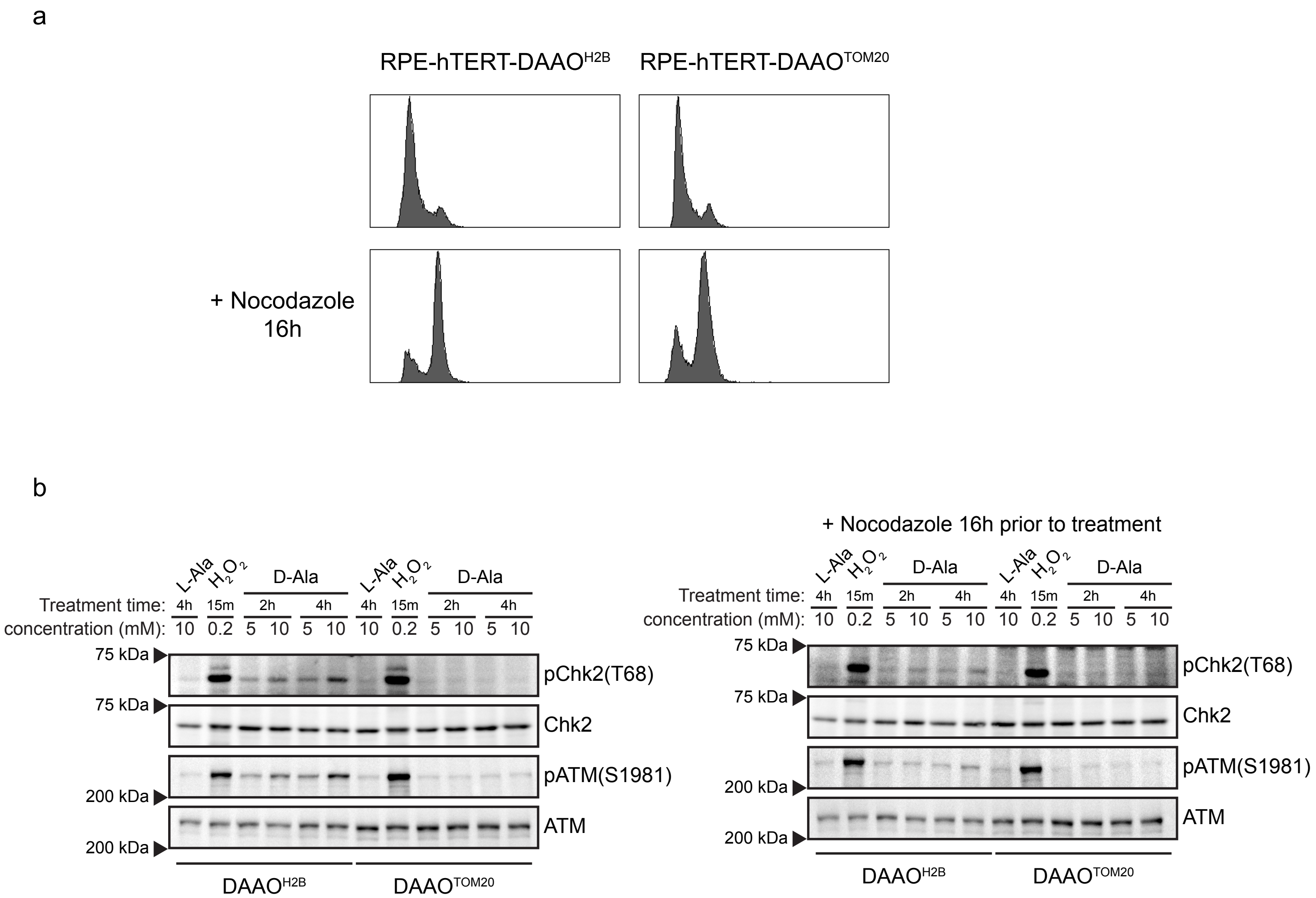

Supplementary figure 8. Mitochondrial H_2O_2 release does not elicit a DNA damage response in mitotic cells. (a) DNA profiles of RPE1-hTERT-DAAO^{H2B} and RPE1-hTERT-DAAO^{TOM20} cells in the absence or presence of nocodazole prior to D-Ala treatment. Nocodazole treatment results in a strong increase of cells with 4N DNA, confirming a mitotic arrest. **(b)** Western Blot for DNA damage response markers in RPE1-hTERT-DAAO^{H2B} and RPE1-hTERT-DAAO^{TOM20} cells treated with L-Ala or D-Ala in de absence or presence of nocodazole. DAAO^{H2B} activation results in ATM and CHK2 phosphorylation regardless of whether cells are blocked in mitosis, while DDR activation is not detected upon activation of DAAO^{TOM20}. Experiment has been repeated several times, 1 biological replicate is shown. Source data are provided in the Source Data file.

Supplementary figure 9 (on next page). Supraphysiological mitochondrial H_2O_2 release in MCF7 breast cancer cells does not induce nuclear DNA damage. (a) Oxygen consumption rate (OCR) upon D-Ala administration as a readout for DAAO activity in monoclonal generated MCF7-DAAO^{H2B} and MCF7-DAAO^{TOM20} cell lines. Experiment was repeated several times with yielding similar results, one biological repeat is shown. Data represent mean values of 7 separate wells -/+ SD. Source data are provided in the Source Data file. **(b)** Normalized OCR values from **(a)** plotted against D-ala concentration, showing that DAAO activity increases proportionally with the D-Ala concentration used. The 3rd timepoint after each D-ala injection is used for this graph. Source data are provided in the Source Data file. **(c)** Measurement of viability of MCF7-DAAO^{H2B} and MCF7-DAAO^{TOM20} cells treated with L- or D-Ala for 48h by PI exclusion. Error bars represent mean values of 3 biological repeats -/+ SD. Per replicate ~10.000 cells were analyzed by Flow cytometry. Source data are provided in the Source Data file. **(d-e)** Western blot of lysates from MCF7-DAAO^{H2B} and MCF7-DAAO^{TOM20} cells treated with D-Ala for several time points. Source data are provided in the Source Data file. **(f)** Quantification of the alkaline comet assay of MCF-DAAO^{H2B} and MCF-DAAO^{TOM20} cells treated with L-Ala or D-Ala for 2h. Graphs display 2 biological repeats for MCF7-DAAO^{H2B} and 3 biological repeats for MCF7-DAAO^{TOM20}; For each repeat 50 comets were analyzed. Horizontal lines represent median values An unpaired two-sided t-test was performed on the averages of the 3 biological repeats of MCF-DAAO^{TOM20} cells. Source data are provided in the Source Data file. ***... continued on next page under figure.***

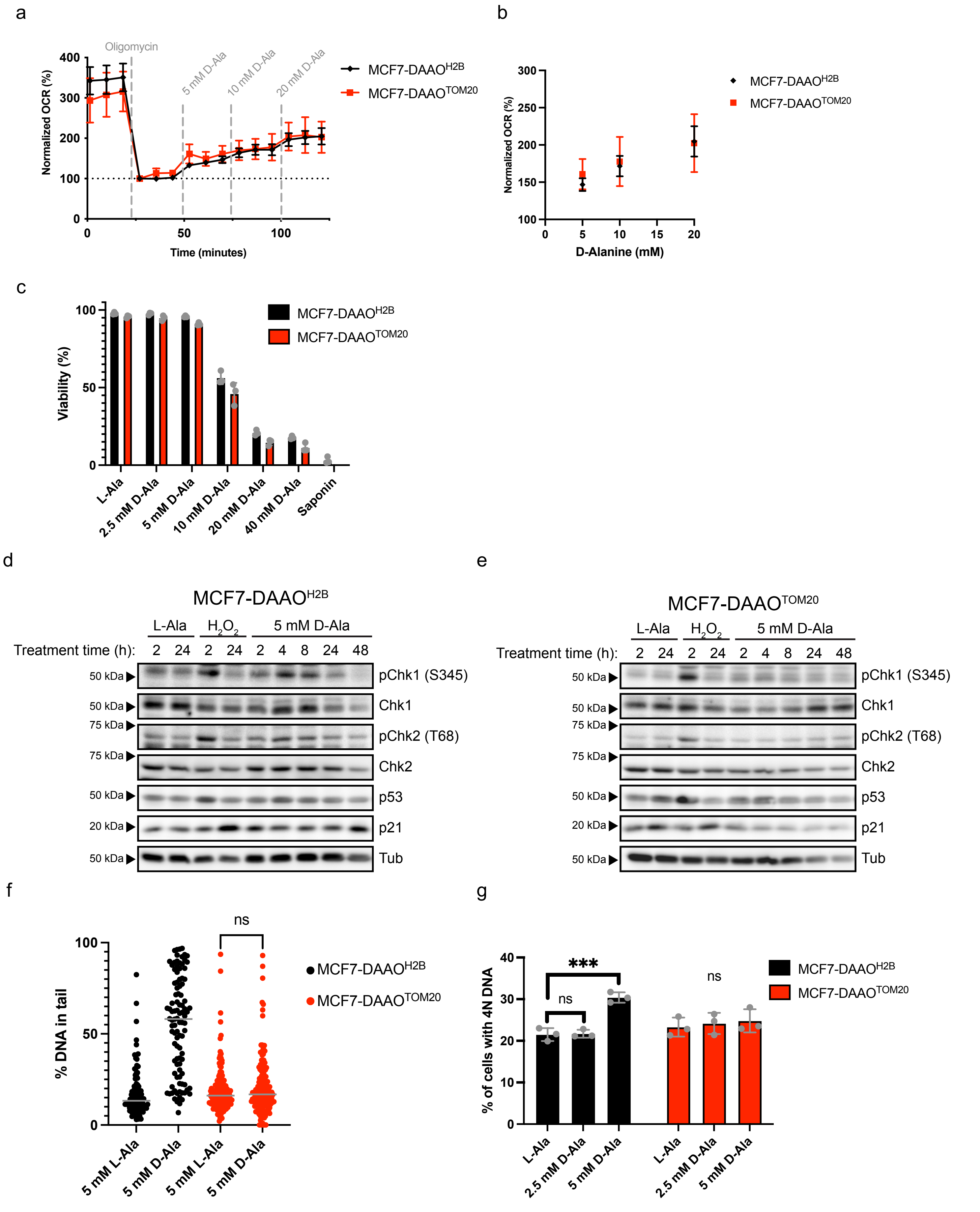

...continued from previous page **(g)** Quantification of cell cycle profiles by flow cytometry, showing the percentage of cells with 4N DNA for MCF-DAAO^{H2B} and MCF-DAAO^{TOM20} cells treated with L-Ala or D-Ala for 48h. Data represent mean values of 3 biological repeats -/+ SD; About 10.000 cells were analysed in each replicate. Statistical significance was determined by one-way ANOVA + Dunnett's multiple comparison test (*** = p≤0.001, DAAO^{H2B} 5 mM D-Ala = 0.003). Source data are provided in the Source Data file.

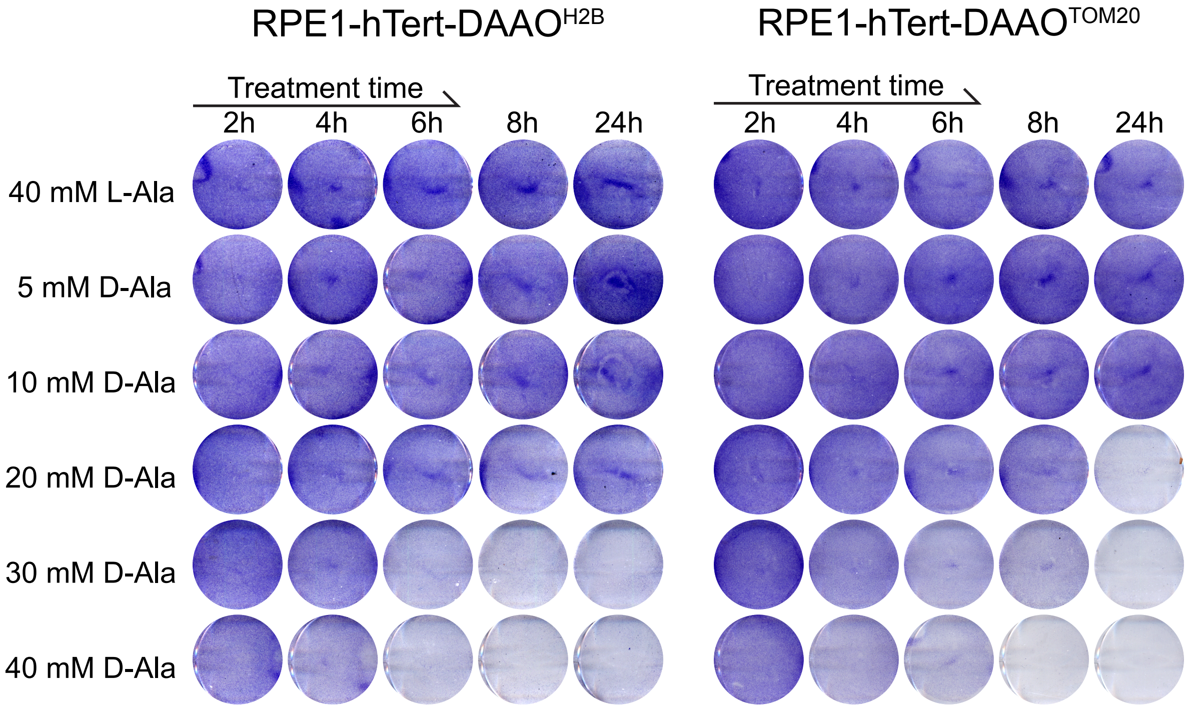

Supplementary figure 10. Viability of DAAO expressing cells is affected by both D-Ala concentration and treatment duration. Crystal Violet staining of RPE1-hTERT-DAAO^{H2B} and RPE1-hTERT-DAAO^{TOM20} treated with several concentrations of L-Ala or D-Ala for different periods. Cells were fixed and stained 24h after treatment was initiated. Viability decreases both by increasing [D-Ala] as well as treatment duration. Experiment has been repeated several times, a typical result is shown.

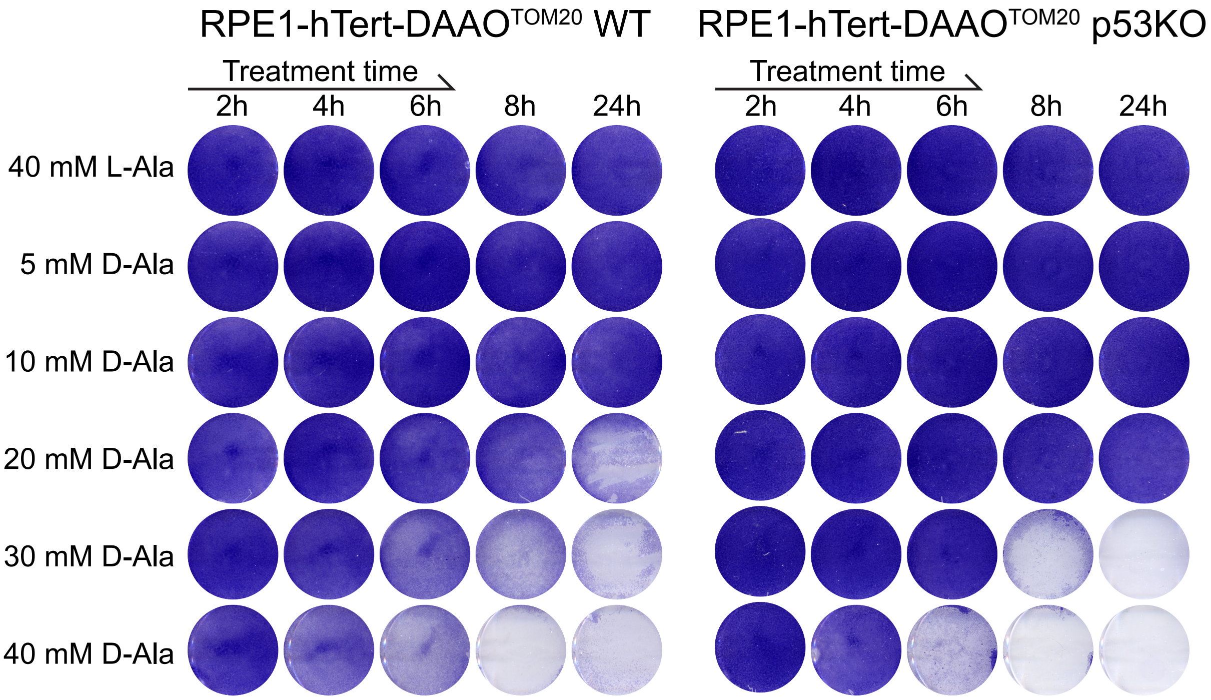

Supplementary figure 11. Oxidative cell death induction in RPE1-hTERT-DAAO^{TOM20} is not p53 dependent. Crystal violet staining of RPE1-hTERT-DAAO^{TOM20} p53 WT and p53 KO cells treated with several concentrations of L/D-Ala for different periods. Cells were fixed and stained 24h after treatment was initiated. Induction of cell death seems to be largely independent of p53 status. Experiment has been repeated several times, a typical result is shown.

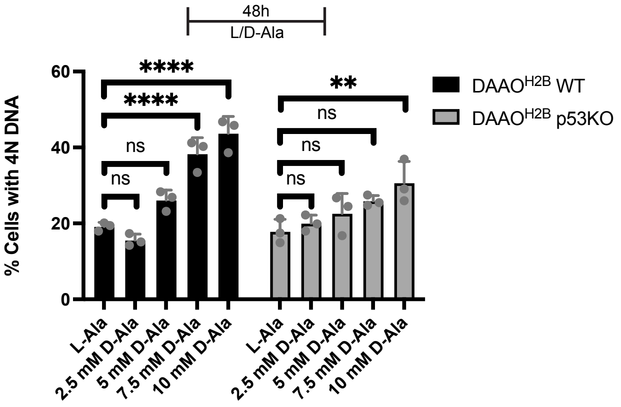

Supplementary figure 12. The 4N arrest induced by activation of DAAO^{H2B} is dependent on p53 expression. Cell cycle profile by PI staining and subsequent Flow cytometry analysis of RPE1-hTERT-DAAO^{H2B} p53 WT and p53 KO cells. Loss of p53 impairs the induction of the tetraploid cell cycle arrest upon DAAO^{H2B} activation. Data represent mean values of 3 biological repeats -/+ SD. For each repeat, ~5000 cells per condition were measured. Statistical significance was determined by one-way ANOVA + Dunnett's multiple comparison test (** = p≤0.01, **** = p ≤ 0.0001, p53 KO 10 mM D-Ala = 0.0082). Source data are provided in the Source Data file.

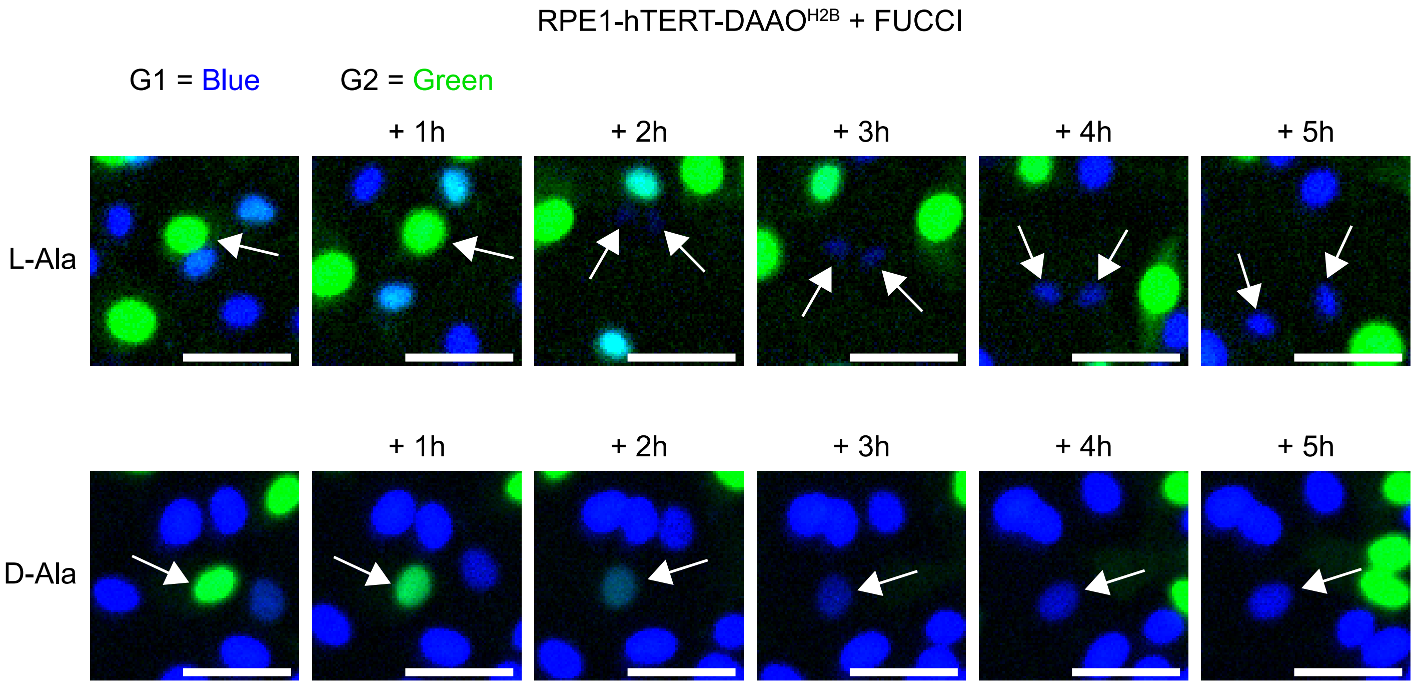

Supplementary figure 13. (cropped stills from Supplementary Movies 1 and 2). RPE-hTERT-DAAO^{H2B} cells expressing the FUCCI cell cycle marker were imaged for 25.5 h in the presence of 10 mM L-Ala (upper panel and Supplementary Movie 1) or 10 mM D-Ala (lower panel and Supplementary Movie 2). Cells in $G_{0/1}$ phase are indicated in blue (mKO2-CDT1) and cells in in S/G_2 phase in green (mAG1-Gemenin). In the presence of L-Ala, RPE1-hTERT-DAAO^{H2B} cells transition normally from G_2 (green) to mitosis (colorless), resulting in the appearance of 2 blue daughter cells. In contrast, addition of D-Ala results in green cells (G2) directly transitioning to blue ($G_{0/1}$) without dividing, indicative of a mitotic bypass. Scale bar = 60 µm.

Supplementary figure 14 (*on next page*). H_2O_2 production at nucleosomes upregulates p21 and downregulates Lamin B1, both hallmarks of senescence. Representative immunofluorescence images of senescence markers of RPE1-hTERT-DAAO^{H2B} and RPE1-hTERT-DAAO^{TOM20} quantified in figure **5d**. LaminB1 decreases and p21 strongly increases upon activation of DAAO^{H2B} but not DAAO^{TOM20}.

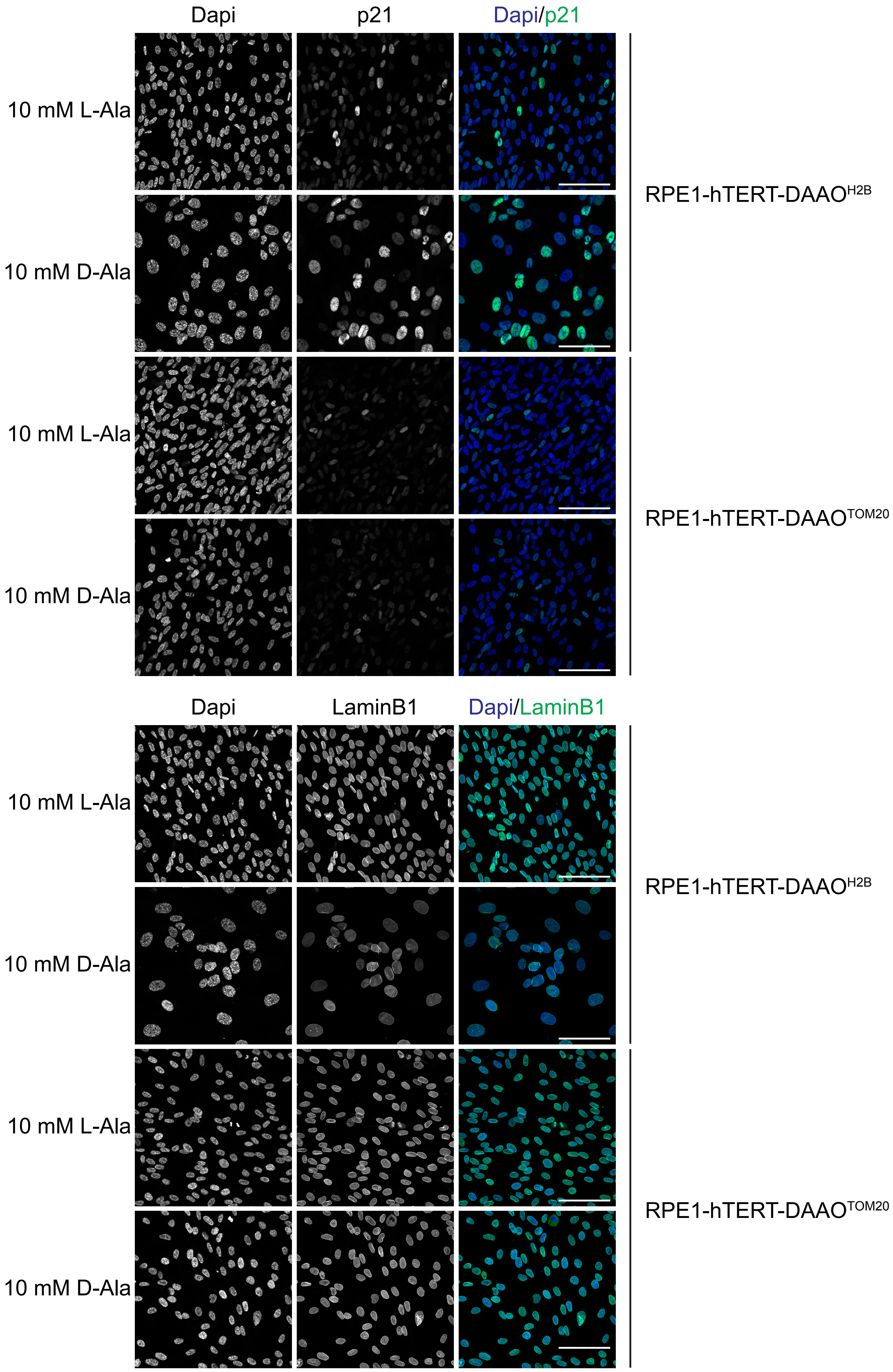

Supplementary Fig 14, ***legends on previous page***

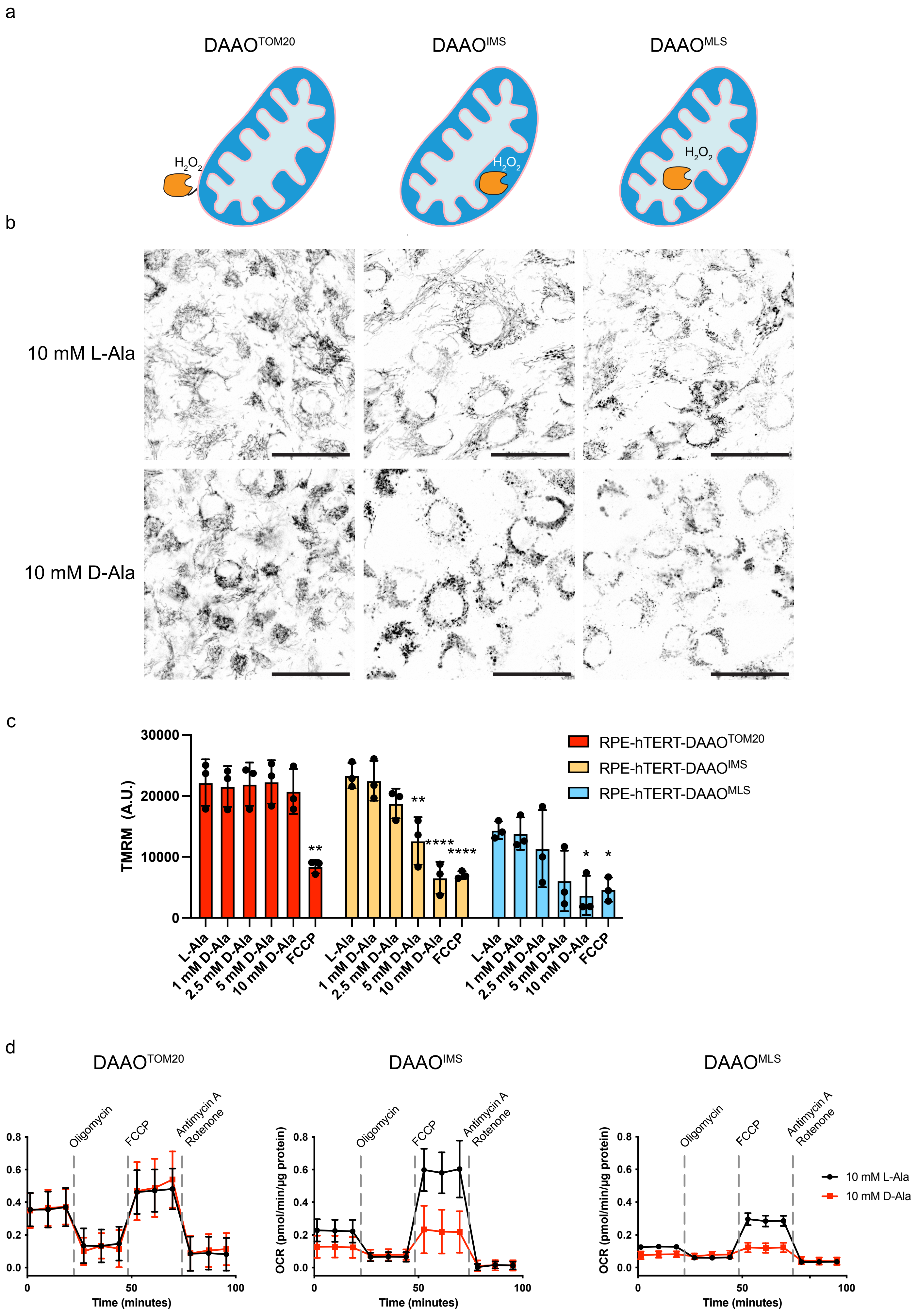

Supplementary Fig 15, *legends on next page*

Supplementary figure 15 (*on previous page*). H_2O_2 production by DAAO^{TOM20} does not damage mitochondrial function, in contrast to intramitochondrial H_2O_2 production. (a) Schematic representation of DAAO localization in RPE-hTERT-DAAO^{TOM20}, RPE-hTERT-DAAO^{IMS} and RPE-hTERT-DAAO^{MLS} lines. **(b)** Representative images of mitochondrial morphology (MitoTracker green, displayed in black on white) in RPE-hTERT-DAAO^{TOM20}, RPE-hTERT-DAAO^{IMS} and RPE-hTERT-DAAO^{MLS} cells treated for 24h with L-Ala or D-Ala. Activation of DAAO^{IMS} and DAAO^{MLS} results in rounding up of mitochondria, indicative of mitochondrial stress. This is not the case for activation of DAAO^{TOM20}. Scale bar = 50 μm. **(c)** Mitochondrial membrane potential as measured by Flow cytometry of TMRM staining of RPE-hTERT-DAAO^{TOM20}, RPE-hTERT-DAAO^{IMS} and RPE-hTERT-DAAO^{MLS} cells treated for 24h with L-Ala or D-Ala. Mitochondrial membrane potential decreases with increasing D-Ala concentration in RPE-hTERT-DAAO^{IMS} and RPE-hTERT-DAAO^{MLS} cells but not hTERT-DAAO^{TOM20} cells. Mitochondrial uncoupler FCCP was added as a positive control. Data represent mean values of 3 biological repeats -/+ SD; ~10.000 cells were measured per replicate. Statistical significance was determined by one-way ANOVA + Dunnett's multiple comparison test (*= p≤0.05 ** = p≤0.01, **** = p ≤ 0.0001, DAAO^{TOM20} FCCP = 0.0011, DAAO^{IMS} 5 mM D-Ala = 0.0016, DAAO^{MLS} 10 mM D-Ala = 0.0201, FCCP = 0.0344). Source data are provided in the Source Data file. **(d)** OCR tracks of the MitoStress test performed on RPE-hTERT-DAAO^{TOM20}, RPE-hTERT-DAAO^{IMS} and RPE-hTERT-DAAO^{MLS} cells pre-treated for 24h with L-Ala- or D-Ala. Both basal (OCR before oligomycin) and maximal (OCR after FCCP) mitochondrial respiration is decreased by pretreatment with 10 mM D-Ala in RPE-hTERT-DAAO^{IMS} RPE-hTERT-DAAO^{MLS} cells but not in RPE-hTERT-DAAO^{TOM20} cells. Experiment was repeated multiple times, one biological replicate is shown. Data represent mean values of 6-7 wells for DAAO^{TOM20} and 3-4 wells for DAAO^{IMS} and DAAO^{MLS} -/+ SD. Data is normalized by protein concentration per well. Source data are provided in the Source Data file.

Supplementary figure 16 (*on next page*). Flow Cytometry gating strategies used in this study. (a) Gating strategy for analyzing DNA content of cells. First a forward scatter (FSC) vs. sideward scatter (SSC) plot is used to gate out cellular debris. Then the area of PI signal (y-axis) is plotted against the width of PI signal (x-axis) to select single cells. The area of PI signal is then plotted in a histogram, and cells with a 2N DNA content an 4N DNA content are quantified. **(b)** Gating strategy for determining cell proliferation by BrdU incorporation. Same strategy as in a was used to remove debris and doublets. Then the signal from the BrdU staining (y-axis) was plotted against the PI staining (x-axis) and BrdU negative and positive cells are quantified. **(c)** Gating strategy to determine cell viability via PI exclusion. First cellular debris is gated out in the same manner as above. Then doublets are removed by plotting the width of FSC (y-axis) against the area of FSC (x-axis). Then a histogram of the PI staining is made and PI negative and PI positive cells are quantified. **(d)** Gating strategy to determine the DNA content of cells that are specifically in $G_{0/1}$ -phase in FUCCI cells. Same strategy as in a is used to remove debris and doublets. Then the green signal (x-axis) and red signal (y-axis) from the FUCCI reporter is plotted to gate cells in $G_{0/1}$. A histogram of the signal from a DNA staining is used to quantify cells with a 2N and 4N DNA content. **(e)** Gating strategy to determine mitochondrial membrane potential by measuring TMRM signal. Same strategy as in c is used to gate out debris and doublets. Then a histogram of the TMRM signal is made and subsequently quantified.

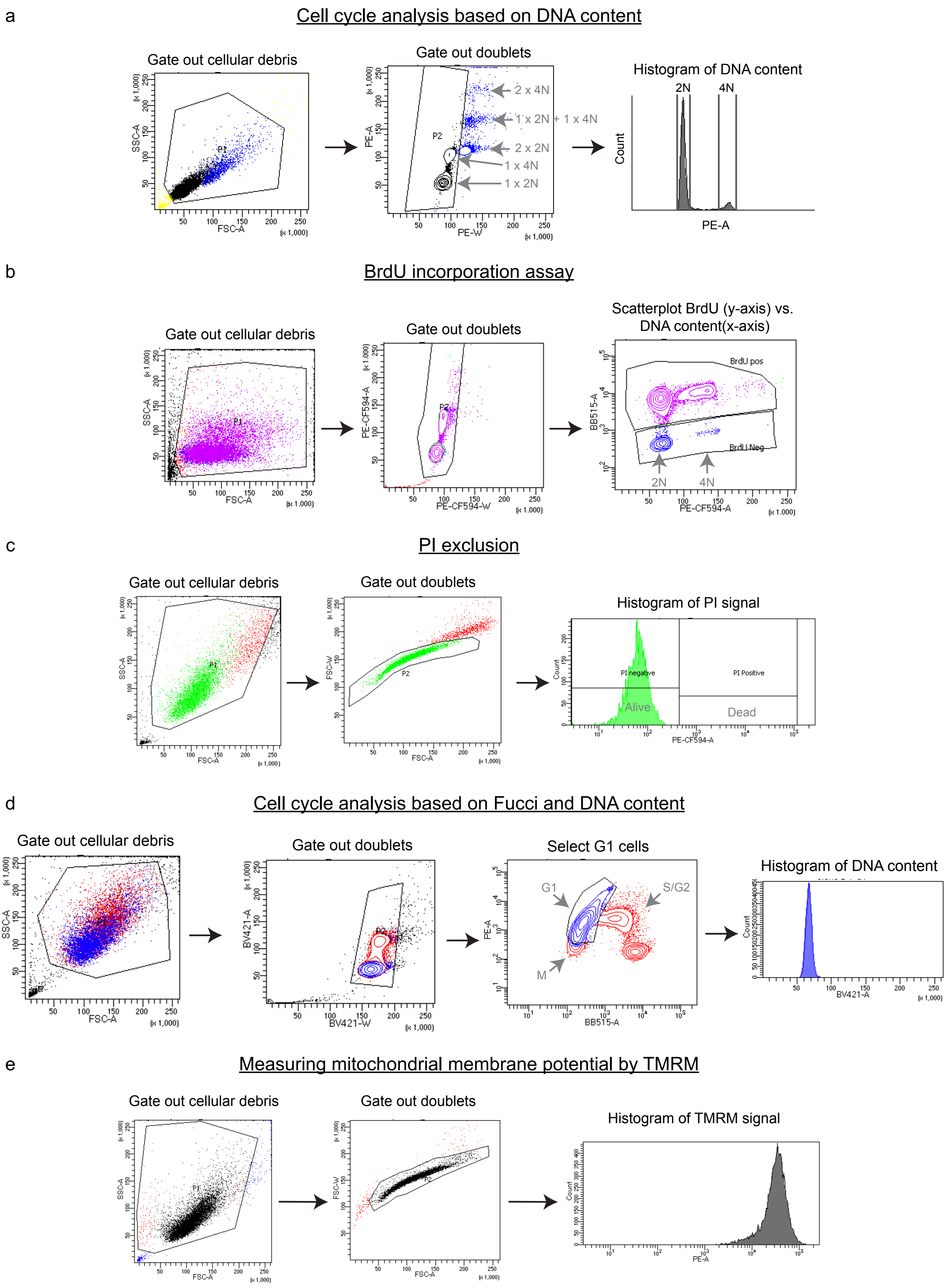

Supplementary Fig 16, *legends on previous page*
