# Supplementary material for: Mitochondrial H2O2 release does not directly cause damage to chromosomal DNA
Source: Nat Commun. 2024 Mar 28;15:2725. doi: 10.1038/s41467-024-47008-x (PMC10978998; doi:10.1038/s41467-024-47008-x)
Supplement: Supplementary file 6 — Reporting Summary [file 41467_2024_47008_MOESM6_ESM.pdf]

nature portfolio

Corresponding author(s): Tobias B. Dansen, PhD

Last updated by author(s): Feb 26, 2024

# Reporting Summary

Nature Portfolio wishes to improve the reproducibility of the work that we publish. This form provides structure for consistency and transparency in reporting. For further information on Nature Portfolio policies, see our Editorial Policies and the Editorial Policy Checklist.

## Statistics

For all statistical analyses, confirm that the following items are present in the figure legend, table legend, main text, or Methods section.

| n/a | Confirmed | |
|---|---|---|
| ☐ | ☒ | The exact sample size (*n*) for each experimental group/condition, given as a discrete number and unit of measurement |
| ☐ | ☒ | A statement on whether measurements were taken from distinct samples or whether the same sample was measured repeatedly |
| ☐ | ☒ | The statistical test(s) used AND whether they are one- or two-sided<br>*Only common tests should be described solely by name; describe more complex techniques in the Methods section.* |
| ☒ | ☐ | A description of all covariates tested |
| ☒ | ☐ | A description of any assumptions or corrections, such as tests of normality and adjustment for multiple comparisons |
| ☐ | ☒ | A full description of the statistical parameters including central tendency (e.g. means) or other basic estimates (e.g. regression coefficient) AND variation (e.g. standard deviation) or associated estimates of uncertainty (e.g. confidence intervals) |
| ☐ | ☒ | For null hypothesis testing, the test statistic (e.g. $F$, $t$, $r$) with confidence intervals, effect sizes, degrees of freedom and $P$ value noted<br>*Give P values as exact values whenever suitable.* |
| ☒ | ☐ | For Bayesian analysis, information on the choice of priors and Markov chain Monte Carlo settings |
| ☒ | ☐ | For hierarchical and complex designs, identification of the appropriate level for tests and full reporting of outcomes |
| ☒ | ☐ | Estimates of effect sizes (e.g. Cohen's *d*, Pearson's *r*), indicating how they were calculated |

*Our web collection on statistics for biologists contains articles on many of the points above.*

## Software and code

Policy information about availability of computer code

| | |
|---|---|
| Data collection | Western Blot, ImageQuant LAS4000 (v1.3); Fluorescence microscopy: Zeiss LSM 880, ZEN 2.3 SP1 (v14.0.20.201); HyPer7 and FUCCI time lapse measurements: Zeiss Cell Observer, ZEN 2.6 Blue (v2.6.76.00000); Seahorse: Wave (v2.6.1.56); Flow Cytometry BD FacsDiva v9.0 |
| Data analysis | Fiji imaging software (v2.9.0/1.54b) was used for analysis of the HyPer7 signal as previously described. Mutational analysis was described in https://pubmed.ncbi.nlm.nih.gov/37719152/<br>Statistical tests and plotting of graphs: Graphpad Prism (v10.11)<br>Flow Cytometry: BD FacsDiva v9.0. |

For manuscripts utilizing custom algorithms or software that are central to the research but not yet described in published literature, software must be made available to editors and reviewers. We strongly encourage code deposition in a community repository (e.g. GitHub). See the Nature Portfolio guidelines for submitting code & software for further information.

## Data

Policy information about availability of data

All manuscripts must include a data availability statement. This statement should provide the following information, where applicable:

- Accession codes, unique identifiers, or web links for publicly available datasets
- A description of any restrictions on data availability
- For clinical datasets or third party data, please ensure that the statement adheres to our policy

All data used to plot the graphs in this manuscript is available in the associated Source Data file. Whole Genome Sequencing data will available from the European Nucleotide Archive https://www.ebi.ac.uk/ena/browser/home under accession number PRJEB72014.

## Research involving human participants, their data, or biological material

Policy information about studies with human participants or human data. See also policy information about sex, gender (identity/presentation), and sexual orientation and race, ethnicity and racism.

| | |
|---|---|
| Reporting on sex and gender | n.a. |
| Reporting on race, ethnicity, or other socially relevant groupings | n.a. |
| Population characteristics | n.a. |
| Recruitment | n.a. |
| Ethics oversight | n.a. |

Note that full information on the approval of the study protocol must also be provided in the manuscript.

# Field-specific reporting

Please select the one below that is the best fit for your research. If you are not sure, read the appropriate sections before making your selection.

☒ Life sciences ☐ Behavioural & social sciences ☐ Ecological, evolutionary & environmental sciences

For a reference copy of the document with all sections, see nature.com/documents/nr-reporting-summary-flat.pdf

# Life sciences study design

All studies must disclose on these points even when the disclosure is negative.

| | |
|---|---|
| Sample size | Sample sizes were chosen in excess to show a significant effect based on pilot experiments used to determine mean and spread. |
| Data exclusions | Experiments in which positive and/or negative controls did not give the expected result were considered invalid and data from these experiments was omitted. |
| Replication | all experiments were replicated as indicated in the figure legends. All experiments in which positive and negative controls showed the appropriate expected result showed similar findings. |
| Randomization | No experiments were performed where samples could or should be assigned randomly to a treatment group. All experiments included appropriate controls seeded from the same batch of cells to compare samples with. |
| Blinding | For experiments where analysis was performed (partially)  manually (e.g. comet assay), investigators were blinded to the sample treatment conditions during analysis. Blinding of Western blot samples makes no sense because it would lead to incomprehensible loading order. |

# Reporting for specific materials, systems and methods

We require information from authors about some types of materials, experimental systems and methods used in many studies. Here, indicate whether each material, system or method listed is relevant to your study. If you are not sure if a list item applies to your research, read the appropriate section before selecting a response.

## Materials & experimental systems

| n/a | Involved in the study |
| --- | --- |
| ☐ | ☒ Antibodies |
| ☐ | ☒ Eukaryotic cell lines |
| ☒ | ☐ Palaeontology and archaeology |
| ☒ | ☐ Animals and other organisms |
| ☒ | ☐ Clinical data |
| ☒ | ☐ Dual use research of concern |
| ☒ | ☐ Plants |

## Methods

| n/a | Involved in the study |
| --- | --- |
| ☒ | ☐ ChIP-seq |
| ☐ | ☒ Flow cytometry |
| ☒ | ☐ MRI-based neuroimaging |

# Antibodies

| | |
| --- | --- |
| Antibodies used | Antibodies used for Western Blot were pChk1 S345 (Cell Signaling, CS2348, lot# 18 1:1000), Chk1 (Santa Cruz, SC8408, lot# A1713 1:1000), pChk2 T68 (Cell Signaling, CS2661, lot# 13 1:1000), Chk2 (Cell Signaling, CS3440, lot# 4 1:1000), ?H2AX (Millipore, 05-636, lot# 3824772, 1:1000), p53 (Santa Cruz, SC-126, lot# E2521 1:2000), p21 (BD Biosciences, 556430, lot# 1173681, 1:1000), ATM (Abcam, ab78, 1:2500) pATM S1981 (Abcam, ab81292, 1:2500) tubulin (Millipore, CP06 OS, lot#3239856 1:2000), PRDX2 (Abcam, ab109367, lot# 1000538-1 1:2000) and PRDXSO2/3 (Abcam, ab16830, lot# GR3294252-3, 1:1000).<br>Antibodies used for IF were LaminB1 (Merck, ZRB1143, lot# Q3250182, 1:500) and p21 (Merck, ZRB1141, lot# 3307439, 1:800). |
| Validation | The antibodies have been used in numerous studies including from our lab (e.g. Shi et al. Fee Radic Biol Med 2021 Aug 20:172:298-311). Positive and negative controls were included in all experiments to show specificity for the tested response. Information regarding specificity and recommended applications can be found on the manufacturers' websites. The antibodies were not used for applications other than listed as suitable on the relevant product pages. |

# Eukaryotic cell lines

Policy information about cell lines and Sex and Gender in Research

| | |
| --- | --- |
| Cell line source(s) | RPE-1-hTert: ATCC cat# CRL-4000, MCF7: ATCC cat# HTB-22, HEK293T: ATCC, cat# CRL-3216 |
| Authentication | Cell lines were authenticated by the ATCC when purchased. Whole genome sequencing confirmed the authenticity of the used RPE1-hTERT parental and p53KO lines. MCF-7 cells were not further authenticated. HEK-293T were only used for lentivirus production and not further analysed. |
| Mycoplasma contamination | All cell lines in our laboratory are routinely checked for mycoplasm. All cell lines used tested negative. |
| Commonly misidentified lines<br>(See ICLAC register) | No commonly misidentified cell lines were used in this study. |

# Flow Cytometry

## Plots

Confirm that:

- ☒ The axis labels state the marker and fluorochrome used (e.g. CD4-FITC).
- ☐ The axis scales are clearly visible. Include numbers along axes only for bottom left plot of group (a 'group' is an analysis of identical markers).
- ☐ All plots are contour plots with outliers or pseudocolor plots.
- ☒ A numerical value for number of cells or percentage (with statistics) is provided.

## Methodology

| | |
| --- | --- |
| Sample preparation | A detailed sample preparation procedure is described in the methods section. |
| Instrument | BD FACSCelesta |
| Software | FACS Diva |
| Cell population abundance | all cells except doublets and debris were included (typically >95% of total events) |
| Gating strategy | Gating strategies for all Flow cytometry experiments are provided in the supplemental figures |

- ☒ Tick this box to confirm that a figure exemplifying the gating strategy is provided in the Supplementary Information.
